# Multiple-sited amyloidosis in the upper aerodigestive tract: case report and literature review

**DOI:** 10.1016/S1808-8694(15)30584-X

**Published:** 2015-10-19

**Authors:** Gustavo Haruo Passerotti, Marcello Caniello, Adriana Hachiya, Patrícia P. Santoro, Rui Imamura, Domingos H. Tsuji

**Affiliations:** 1 Resident physician.; 2 Resident physician.; 3 Preceptor.; 4 PhD. Collaborator Physician.; 5 PhD. Assistant Physician.; 6 Associate Professor. Work carried out at the Otorhinolaryngology Department - University of São Paulo Teaching Hospital.

**Keywords:** amyloidosis, waldeyer's ring, dysphagia, larynx, rhinopharinx

## Abstract

There are some reports of localized amyloidosis in the larynx, an entity that corresponds to one percent of all benign tumors of this region. However, there are only two cases of amyloidosis in the Waldeyer's ring 6, 13, 14. We hereby describe a rare case of amyloidosis in areas not associated with the upper aero-digestive tract: tonsil pillar, rhinopharynx, supraglottis and glottis, without visible continuity of amyloid tissue. We will also discuss post-operative follow up with severe dysphagia.

## INTRODUCTION AND LITERATURE REVIEW

Laryngeal amyloidosis accounts for about 1% of all laryngeal benign tumors, more prevalent in males at a 3:^1^ ratio, more often at their 5th decade of life^2^. It is usually localized, primary (idiopathic), AL type and, very rarely, it is followed by a systemic infection^2^.

It may manifest as a systemic disease, or limited to certain organs resulting from the extracellular deposits of amyloid protein.

There are about 20 different types of amyloid protein; however, despite subtle differences in aminoacid sequences, all of them present similar spatial orientation (β-folded), providing them with the quality of being fibrous, insoluble and resistant to proteolysis.

They can be primary disorders (with localized or systemic involvement), secondary to infectious processes such as tuberculosis, leprosy and osteomyelitis or to chronic inflammatory processes such as rheumatoid arthritis (both systemic and localized involvement), or, still, secondary to post-hemodialysis such as it happens in amyloidosis caused by β2 microglobuline.

There is also classification according to the type of amyloid protein such as: light chain (LC), type A (AA), type β2 microglobuline (Aβ2M), heavy chain (HC), among others.

Classifications overlap. For example, LC encompasses the primary idiopathic in which there is a monoclonal increase in γ globuline without apparent cause and it also involves the one that is secondary to multiple myeloma. It has worse prognosis, because it is more often associated with systemic disorders, and among the most severe ones we have the cardiac, rarely AA type. Notice that, although laryngeal amyloidosis is of the LC type, it is usually localized.

The following report is a peculiar case thanks to, especially, to two aspects: the rarity and multiplicity of the lesions, and also, the difficulty in treating the severe dysphagia that characterized this case.

## CASE REPORT

Female, 55 years old, Caucasian, complaining of floating hoarseness, mild respiratory discomfort and dysphagia, especially for grains, lasting for 8 years and with important worsening in the last 3 months. She brought with her the results from a pathology exam done in another center that suggested amyloidosis. As comorbidities she had systemic arterial hypertension, angina and epigastralgia.

**Figure 1:**
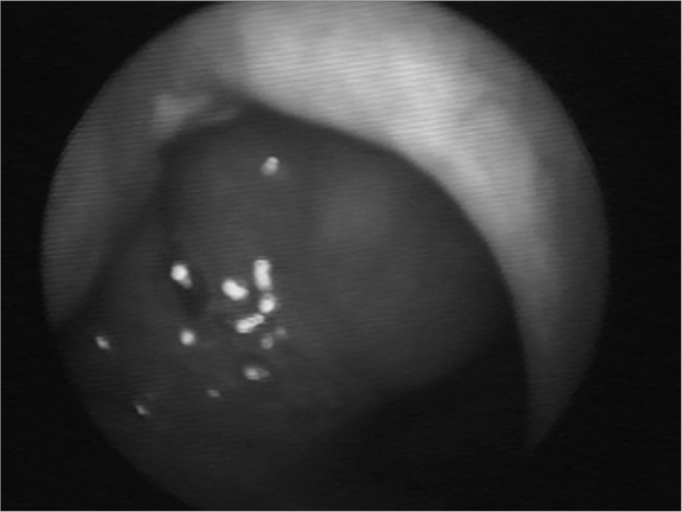
Amyloid lump aspect on the right sid-e of the rhinopha-

**Figure 2:**
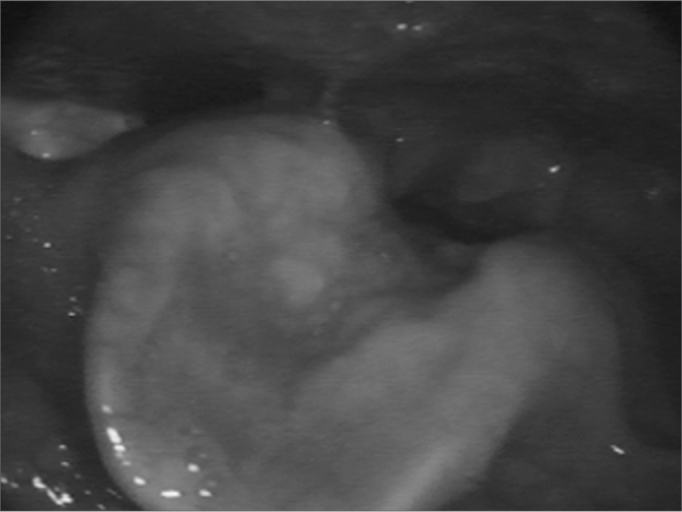
Epiglottis infiltrated by the amyloid substance.

**Figure 3:**
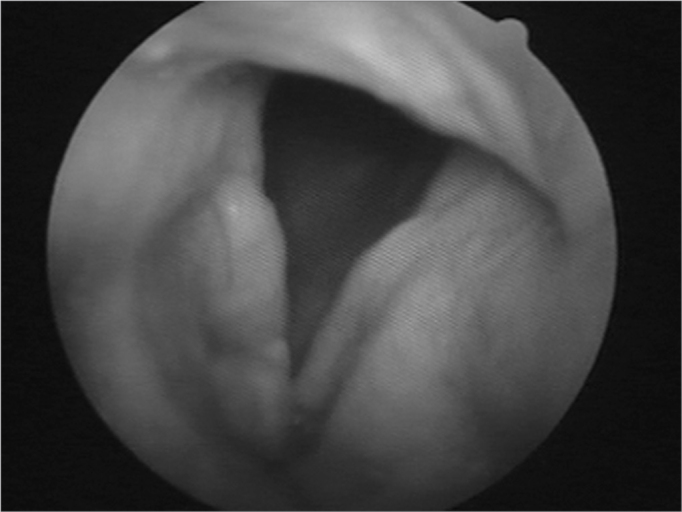
Amyloidosis involving the right vocal fold.

Nasofibroscopy exam revealed multiple masses: in the rhinopharynx, posterior tonsil lateral wall, laryngeal face of the epiglottis, ventricular folds, aryepiglottic folds, right vocal fold (Figures 1, 2 and 3). CT scan of the region showed localized lesions. We investigated the patient in order to rule out systemic disease, she had normal renal (clearance and proteinuria) and liver function, fasting glucose, CBC, protein electrophoresis, rheumatoid factor, anti-nucleus factor and PPD, all normal. Digestive endoscopy was also normal. She underwent chest X-ray, which showed an enlarged heart area because of an enlarged left ventricle (LV). Her echocardiogram showed mitral regurgitation and diffuse involvement of the left ventricle, in a moderate degree. Her EKG showed left branch block. She was evaluated by a cardiologist, who diagnosed coronary disease, which could be treated after excision biopsy of the amyloid masses through nasal endoscopy and suspension laryngoscopy.

The patient was then taken to the operating room and the rhinopharynx mass was partially removed with a cutting forceps in order to avoid a large open wound and later local stenosis. The masses in the posterior tonsillar pillar, in the epiglottis, right ventricular fold, and that in the right vocal fold were all removed by laser. We also carried out a tracheotomy in order to secure the airway.

In the 2nd post-operative day (2PO), we observed food residues oozing out of the tracheal cannula. We then decided for a diet through the nasogastric tube (NGT). The patient developed a bronchopneumonia because of aspiration and was started on proper antibiotics. On the 7th PO a new test was carried out and we noticed the oozing of food through the NGT. Since she was breathing normally when the cannula was plugged, we decided to remove the cannula. However, even receiving her food through the NGT, she still aspirated. We carried out a function assessment of her swallowing, a swallowing videoendoscopy (SVE) test with thick and pasty-like liquid food per os, and there still was residue left after swallowing, laryngeal food penetration during swallowing, followed by laryngotracheal aspiration and cough reflex. We diagnosed a severe mechanic oropharyngeal dysphagia, with stiffening of the entire supraglottic structures, compromising the efficacy of laryngeal protection mechanisms during swallowing (especially epiglottis retro-version and shortening of the aryepiglottic fold), causing the laryngotracheal food aspiration. She was referred to a speech therapist for swallowing rehabilitation, with emphasis in supraglottic maneuver exercises (holds the breath, swallows, cough and swallows again), and forced swallowing.

**Figure 4:**
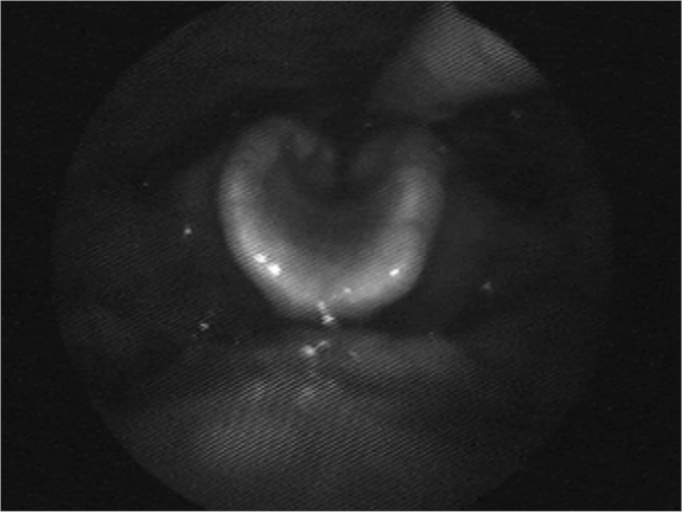
Swallowing videoendoscopy showing dye penetration in the laryngeal vestibule.

**Figure 5:**
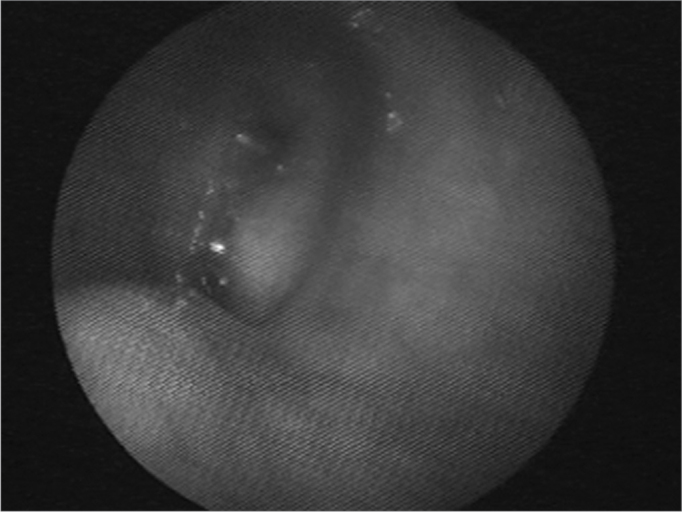
Swallowing videoendoscopy under subglottic vision confirming laryngotracheal liquid aspiration.

The patient was doing well in the post-operative; she started to feed by mouth with the therapist support, swallowing only pasty food, still avoiding liquids and solids. Nonetheless, she presented with acute respiratory failure, and it was necessary to perform an emergency tracheotomy, causing an important worsening in her swallowing, and she returned to food intake through the nasogastric tube. She was refereed to a new SVE one month after the emergency tracheotomy, still feeding through the NGT only. We tested liquid, thick liquid and pasty food, and there was penetration of all of them (Figure 4). She underwent food intake with subglottic visualization, and there was liquid aspiration, with good response to the supraglottic maneuvers (Figure 5). Thick liquid or pasty food were not aspirated. She was then diagnosed with severe oropharyngeal dysphagia for liquids, and instructed to intensify her swallowing therapy with daily sessions in order to remove the NGT.

The patient followed the instructions properly and really helped out during the sessions, and she improved. A new SVE after one week under subglottic - conventional vision did allow proper visualization of the glottic slit and, consequently, of laryngotracheal aspiration. We did not observe aspiration with any of the food types during the test, although there was contrast spill over the laryngeal vestibule, above the vocal folds. She was diagnosed with moderate oropharyngeal dysphagia, with good compliance with the supraglottic maneuver, and this allowed us to remove the NGT and to keep her feeding per os.

## DISCUSSION

In the head and neck, amyloidosis may involve the nose^3^, paranasal sinuses^3^, ^4^, nasopharynx, oropharynx, tonsils^5^, ^6^, oral cavity, tongue, tracheobronchial tree and larynx. The larynx is the most usually involved site, and according to Mitrani^7^, in a descending order of prevalence, it involves: ventricular folds (55%), laryngeal ventricle (36%), subglottic space (36%), vocal folds (27%), aryepiglottic folds (23%) and anterior commissure (14%). Macroscopically it may be a diffuse subepithelial edema without mucosa (more common) or nodular (less frequent)^1^, ^2^ alterations.

Amyloidosis of the upper aerodigestive tract is usually localized^1^, ^8^, however tongue involvement^2^, ^9^ is an exception and is very much associated with the systemic disease.

It is frequent to have multiple laryngeal sites or, even, that of the aerodigestive tract^1^, ^9^, ^10^ and tongue and trachea are the ones most frequently associated with laryngeal amyloidosis and, in such cases, systemic involvement is also more common.

It is imperative that the otorhinolaryngologist, when facing a diagnosis of aero-digestive tract amyloidosis, understand the need for systemic investigation. In order to investigate systemic diseases, we need tests that help us assess the function of possible target organs, such as the kidneys, liver, lower respiratory tract, digestive tract, heart and also tests that track down chronic systemic disorders such as multiple myeloma, tuberculosis and rheumatoid arthritis. Fine needle aspiration of the subcutaneous abdominal fat is the exam of choice to detect systemic amyloidosis^11^.

Our patient had heart symptoms; however, all the tests told us she did not have amyloid heart infiltration, but rather a coronary disorder secondary to chronic systemic hypertension.

Our patient complained of dysphonia, dyspnea and dysphagia. She already had had dysphagia for some time; however it worsened in the post-operative, very likely because of post-operative scar fibrosis, with stiffening and changes in the mechanisms that protect the supraglottis and also the tracheostomy cannula that prevents the mechanical movement of effective laryngeal elevation and anteriorization to control dysphagia.

In the literature, the most reported symptom of patients with laryngeal amyloidosis is hoarseness^1^, and dysphagia^12^, common in cases of amylolytic macroglossia, rarely described^2^, ^5^.

Our case is the 4th report of amyloidosis in the Waldeyer's ring. Beiser^6^ described one case of amyloidosis in the tongue base, tonsils and rhinopharynx. Simpson described a case of amyloidosis in the rhinopharynx only, causing Eustachian Tube dysfunction^13^. Green^14^ reported recurrent amyloidosis in many distinct laryngeal sites, such as the tonsils, tongue base, rhinopharynx and supraglottis. Therefore, we have here the first case in which there is simultaneous involvement of the larynx and rhinopharynx.

Diagnosis is carried out through biopsy in the suspected organ (Congo Red Dye under polarizing light microscopy). It can also be confirmed by the negative potassium permanganate proteolysis test.

In the case of symptomatic and localized amyloidosis, as it usually happens in cases of laryngeal amyloidosis, the lesion must be removed and the patient must be started on periodic treatment, because local and distant relapses are common^1^, ^14^. More over, one must be in constant look out for systemic lesions.

Treatment is based on surgical resection. As it happened in our case, in some cases, tracheostomy is necessary in order to secure the airway^1^.

The prognosis for laryngeal amyloidosis is excellent if the lesions are completely removed. In certain cases (asymptomatic) the mass can be seen, in other words, clinical follow up.

## FINAL REMARKS

Laryngeal amyloidosis is a differential diagnosis among the lumps and lesions in the upper aero-digestive tract and its location is diverse, in some cases, multiple. We must also bear in mind that the lesion may be a consequence or be associated with some systemic diseases, which must be treated in order to prevent amyloidosis from becoming systemic. Dysphagia is a symptom that, although rare, may happen in amyloidosis patients, often times indicating failure in laryngeal competence against laryngotracheal aspiration.
